# Forensic dental age estimation with deep learning: a modified xception model for panoramic X-Ray images

**DOI:** 10.1007/s12024-025-00962-4

**Published:** 2025-02-12

**Authors:** Ercument Yilmaz, Cansu Görürgöz, Hatice Cansu Kış, Emin Murat Canger, Bengi Öztaş

**Affiliations:** 1 Department of Software Development, Karadeniz Technical University, Trabzon, Türkiye; 2 Department of Computer Technologies, Karadeniz Technical University, Trabzon, Türkiye; 3 Yılmaz Bilişim R&D Consulting Software Engineering and Services Trade Limited Company, Trabzon, Türkiye; 4 Department of Dentomaxillofacial Radiology, Faculty of Dentistry, Bursa Uludağ University, Bursa, Türkiye; 5 Department of Orthodontics, Faculty of Dentistry, Tokat Gaziosmanpasa University, Tokat, Türkiye; 6 Department of Oral and Maxillofacial Radiology, Faculty of Dentistry, Erciyes University, Kayseri, Türkiye; 7 Department of Dentomaxillofacial Radiology, Faculty of Dentistry, Ankara University, Ankara, Türkiye

**Keywords:** Forensic diagnostic, Dental age estimation, Minimum age of criminal responsibility, Deep learning, Xception

## Abstract

**Purpose:**

This study aimed to develop an improved method for forensic age estimation using deep learning models applied to orthopantomography (OPG) images, focusing on distinguishing individuals under 12 years old from those aged 12 and above.

**Methods:**

A dataset of 1941 pediatric patients aged between five and 15 years was collected from two radiology departments. The primary research question addressed the identification of the most effective deep learning model for this task. Various deep learning models including Xception, ResNet, ShuffleNet, InceptionV3, DarkNet, NasNet, DenseNet, EfficientNet, MobileNet, ResNet18, GoogleNet, SqueezeNet, and AlexNet were evaluated using traditional metrics like Classification Accuracy (CA), Sensitivity (SE), Specificity (SP), Kappa (K), Area Under the Curve (AUC), alongside a novel Polygon Area Metric (PAM) designed to handle imbalanced datasets common in forensic applications.

**Results:**

“Forensic Xception” model derived from Xception outperformed others, achieving a PAM score of 0.8828. This model demonstrated superior performance in accurately classifying individuals’ age groups, with high CA, SE, SP, K, AUC, and F1 Score. Notably, the introduction of the PAM metric provided a comprehensive evaluation of classifier performance.

**Conclusion:**

This study represents a significant advancement in forensic age estimation from OPG images, emphasizing the potential of deep learning models, particularly the “Forensic Xception” model, in accurately classifying individuals based on age, especially in legal contexts. This research suggests a promising avenue for further advancements in forensic dental age estimation, with future studies encouraged to explore additional datasets, refine models, and address ethical and legal considerations.

## Introduction

The determination of the age of criminal responsibility for children is an issue of global importance and debate, based on principles of justice, human rights and child protection. The United Nations Convention on the Rights of the Child (CRC) [1], which has been endorsed by most nations, provides guidelines for safeguarding the rights and best interests of children, including their responsibility within the criminal justice system.

The minimum age of criminal responsibility varies widely between countries. As a result, this is a complex issue that needs to be carefully analyzed. The CRC recommends that states raise their minimum age to meet international standards, especially if it is below 12 years of age [1]. Many countries, including China, Indonesia, Brazil, Egypt, Turkey, the United Kingdom, Canada, the Netherlands and Belgium, have adjusted their minimum age of criminal responsibility (MACR) to align with the CRC guidelines, representing approximately 34% of the world’s population [2, 3]. This adjustment demonstrates their commitment to upholding the rights and best interests of children in accordance with international standards.

In Turkey, juvenile criminal responsibility is regulated by specific age categories [4]. According to Turkish law, individuals under 12 years old are not held criminally responsible for their actions, with protective measures available for serious offenses. This approach is in line with Turkey’s CRC obligations and ensures fair treatment and protection of children in the criminal justice system.

Forensic odontology (FO) is essential for human identification, with dental age estimation (DAE) being a key technique. The 2009 US National Academy of Sciences (NAS) report highlighted the need for extensive research in FO [5]. A systematic review from 2014 to 2019 identified 644 studies, with DAE accounting for 41.3% of the research. This focus on DAE responds to modern challenges such as migration and determining the age of criminal responsibility. Systematic reviews have shown an increasing emphasis on DAE within FO [6].

DAE plays a central role in FO for human identification. Recent research focused on improving DAE methods by integrating traditional expertise with machine learning and deep learning (DL) models, significantly improving accuracy and expanding applications in clinical dentistry, legal proceedings and healthcare.

Studies have compared the effectiveness of the Willems and Cameriere methods in the Turkish population. A study updated Cameriere’s regression equation for Turkish adolescents and found that Cameriere’s method provided closer estimates to chronological age (CH), whereas Willems’ consistently overestimated dental age [7]. Further research in southern Turkey confirmed a significant correlation between DA and CH in girls [8]. Another study [9] found that Willems and the London Atlas were the most accurate methods for Turkish children. A novel formula combining dental and skeletal parameters showed a 72.80% success rate in Turkish children, outperforming existing models [10].

Some studies have found limitations of existing methods in different populations, such as in New Zealand [11]. Alternative methods like the Kvaal method have been suggested as a solution [12]. A Brazilian review recommended correction factors for age estimation [13]. Demirjian’s method showed variable accuracy in Brazilian children [14].

Machine learning and deep learning techniques have been increasingly applied to improve DAE. Studies using convolutional neural networks (CNNs) and other machine learning algorithms have outperformed traditional methods in predicting DA, showing higher accuracy rates in different populations [14–27]. For example, CNNs have estimated DA from panoramic radiographs for individuals aged 10–25 years, with promising results [15]. In forensic and clinical dentistry, Demirjian’s method with a fuzzy neural network accurately classified the age of South Indian children aged 4 to 18 years [16]. A study found that a CNN model using OPG images was more accurate for age cut-offs at 14, 16 and 18 years [17]. Machine learning algorithms outperformed traditional methods in predicting DA in French patients [18]. Specific architectures, such as CVIP-Net [28], SN-CNN [29] and Xception [30], have demonstrated high accuracy in age and sex identification using OPG images.

Research has aimed to improve and validate DAE methods using new techniques and technologies. Combining traditional expertise with machine learning has improved accuracy in clinical dentistry, legal proceedings and healthcare. Integrating AI with traditional DAE methods improves accuracy and efficiency across different populations and applications. Further research is needed to develop reliable age estimation systems using radiological images [31].

In this study, we explore the intersection of FO and DL, representing a notable advance in DAE. Our investigation uses established DL techniques and a custom Xception model, ‘Forensic Xception’, for binary age classification. This approach improves accuracy, reliability and addresses child protection, justice and human rights issues. Our research offers a fresh perspective on how DL can navigate age classification in the context of criminal responsibility.

## Materials and methods

### Deep learning models

This study evaluates 17 deep learning (DL) models from the literature for determining criminal responsibility based on age, focusing on the critical 12-year-old threshold. The best-performing model was then modified to create the 18th model, which is the primary contribution of this work.

Selecting an appropriate model requires considering both the problem domain and the desired performance. Over the past decade, DL architectures have revolutionized computer vision and image analysis, each offering unique design principles, computational strategies, and application areas. Understanding these influential models helps in identifying the most suitable approach for dental age estimation (DAE).

Early milestones in DL include architectures like AlexNet [32], which introduced convolutional layers and ReLU activation functions in an 8-layer network, setting a high performance benchmark. Building on these foundations, GoogleNet’s Inception architecture [33] incorporated multiple convolutional filter sizes within the same module, achieving exceptional accuracy with a 22-layer depth. Around the same time, ResNet [34] tackled the vanishing gradient problem through residual blocks, enabling the successful training of very deep networks and resulting in versatile variants like ResNet-18, ResNet-50, and ResNet-101.

Subsequent innovations addressed efficiency, scalability, and specialized tasks. Darknet [35], serving as the backbone of YOLO, emphasizes real-time object detection with GPU acceleration. SqueezeNet [36] significantly reduces parameters while maintaining accuracy, making it ideal for mobile and edge devices. InceptionV3 [37] refines the Inception concept to facilitate transfer learning in fields like medical imaging, while Inception-ResNetV2 [38] merges the strengths of Inception and residual connections to boost accuracy further.

Later contributions focused on computational efficiency and architectural search. Xception [39] employs depthwise separable convolutions for improved performance at lower computational cost, and DenseNet-201 [40] leverages dense connections between layers to combat vanishing gradients and reduce redundancy. MobileNetV2 [41] and ShuffleNet [42] address resource constraints by optimizing for mobile and embedded environments, employing techniques like inverted residuals, depthwise separable convolutions, group convolutions, and channel shuffling. NasNetLarge and NasNetMobile [43], generated via Neural Architecture Search, dynamically balance efficiency and performance. Finally, EfficientNetB0 [44] scales network depth, width, and resolution in a principled manner to deliver a strong balance of speed and accuracy.

These architectures collectively highlight the wide range of deep learning techniques applicable to image-based tasks. From foundational advancements in network depth and activation functions to cutting-edge innovations in model optimization and parameter tuning, they provide valuable insights for selecting the most effective approach to DAE in forensic investigations.

### Forensic xception deep learning model

**Fig. 1:**
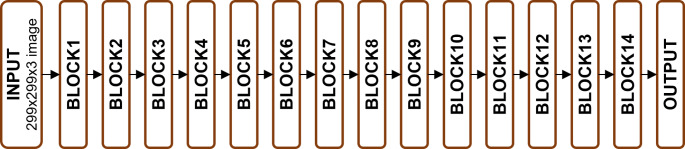
Xception model diagram

Xception, short for “Extreme Inception,” is a deep CNN architecture that balances computational efficiency with high performance in image classification (Fig. 1). Its core innovation lies in depthwise separable convolutions, which split standard convolutions into depthwise and pointwise operations, reducing computational costs and parameter counts.

**Fig. 2:**
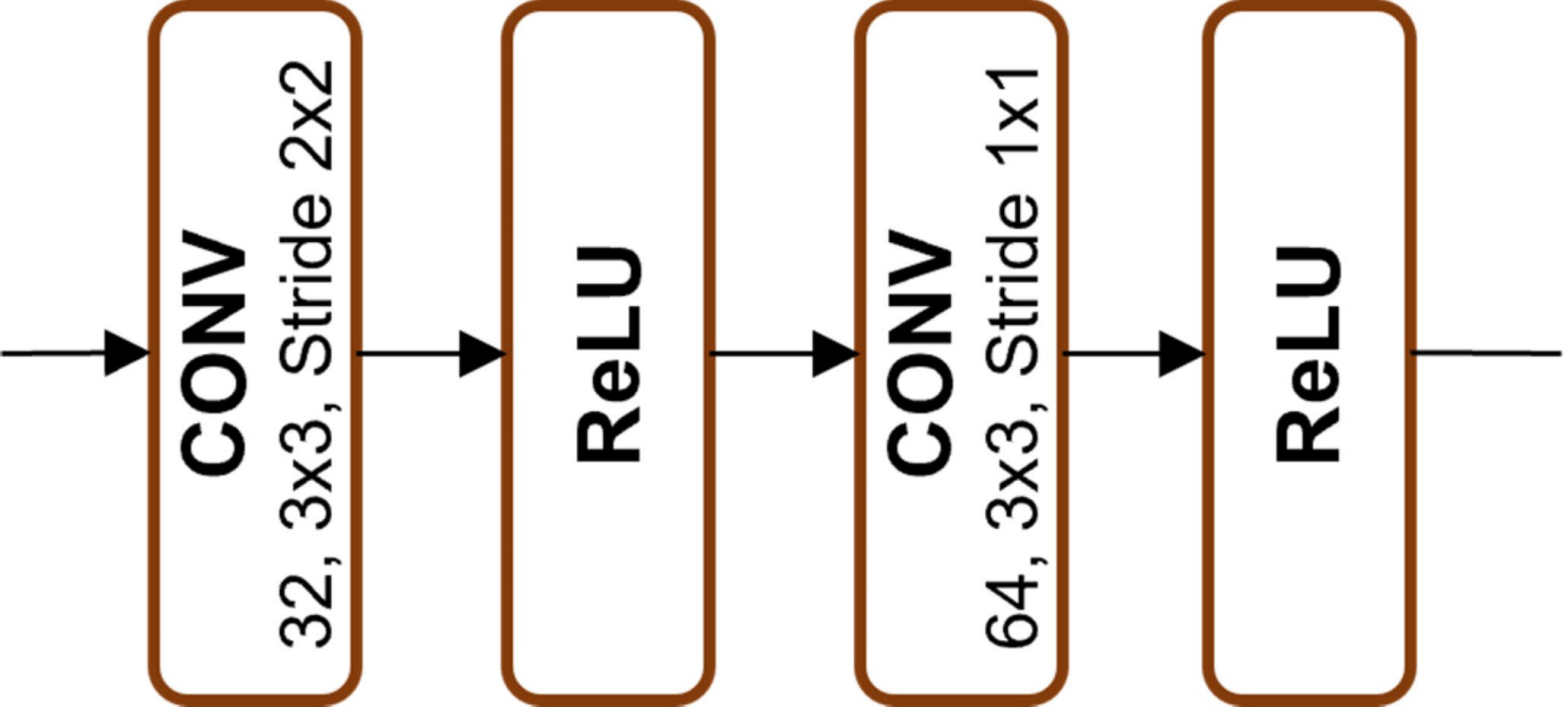
Block 1

**Fig. 3:**
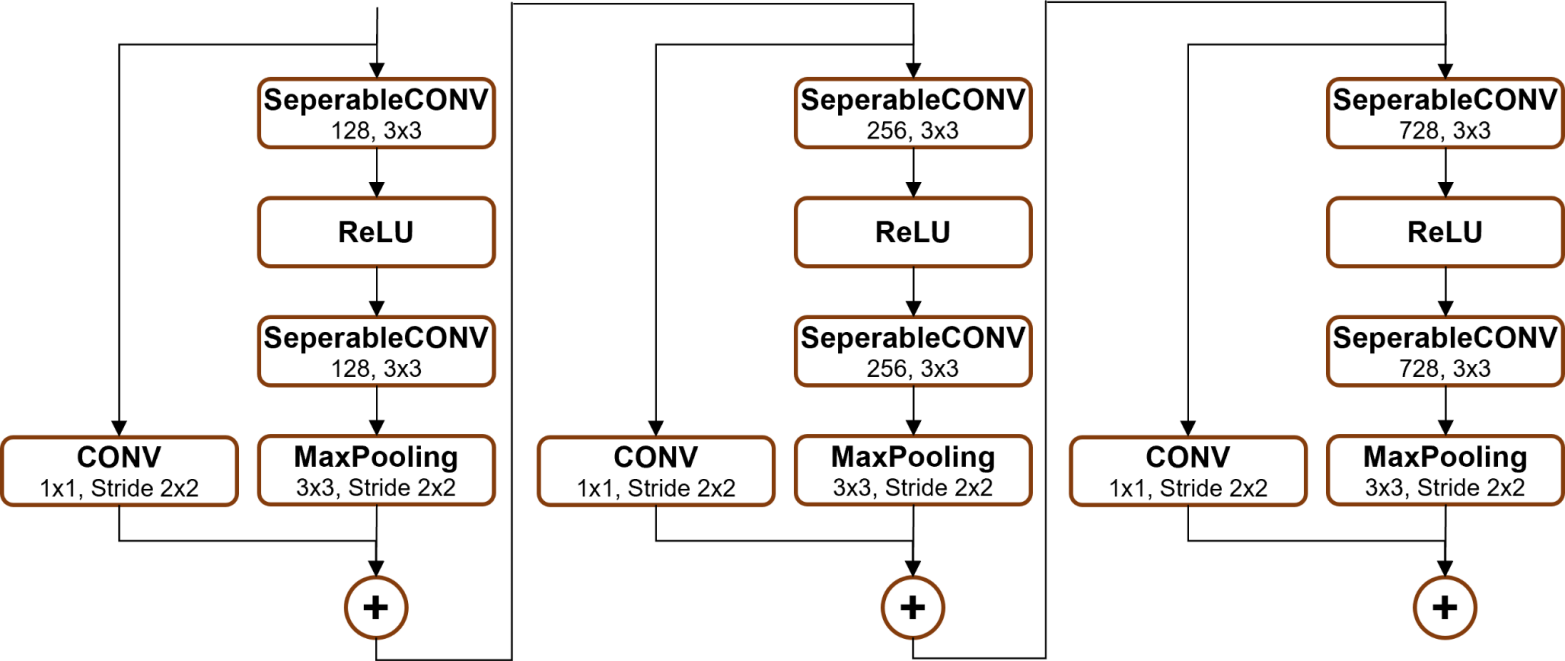
Blocks 2, 3 and 4

**Fig. 4:**
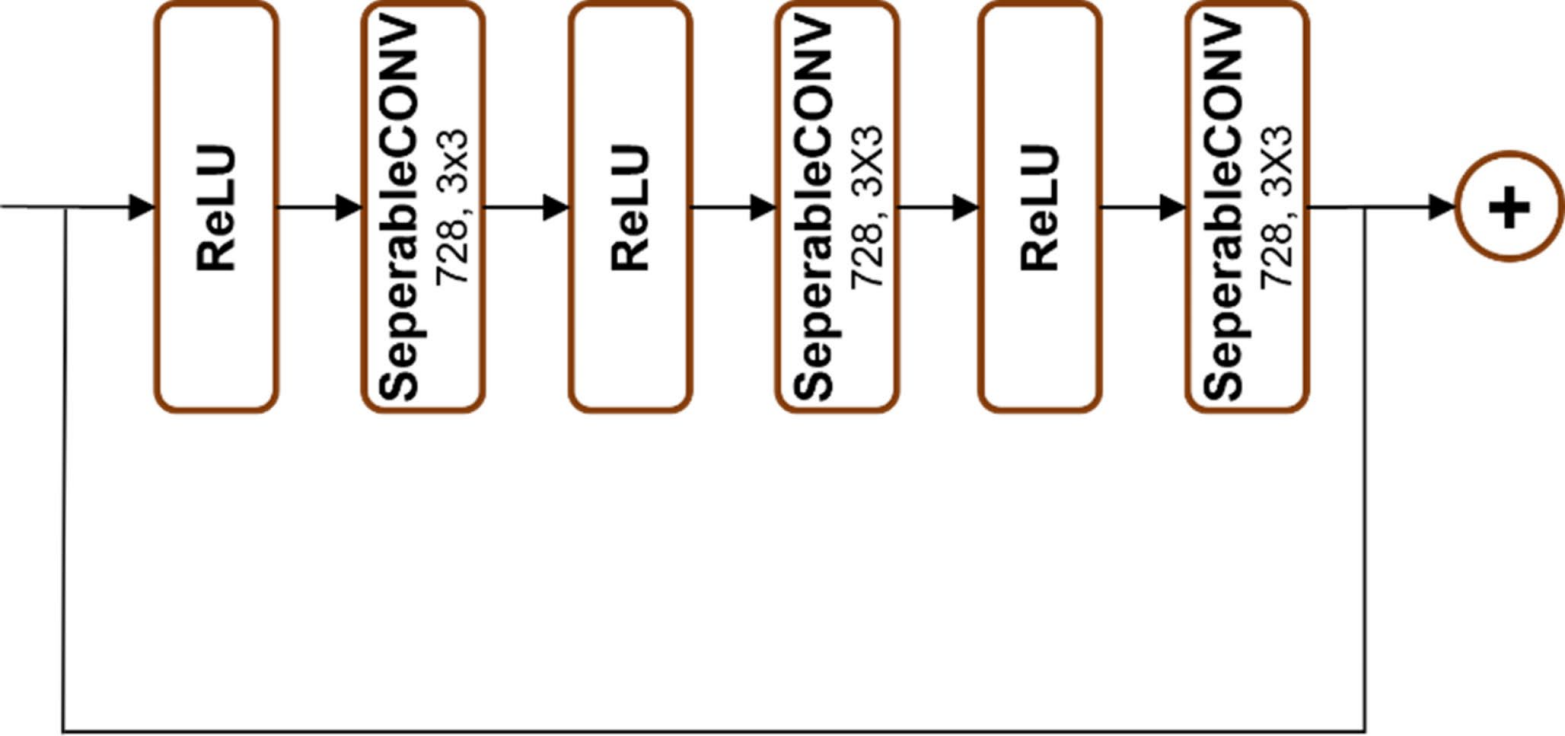
Blocks 5, 6, 7, 8, 9, 10, 11 and 12

**Fig. 5:**
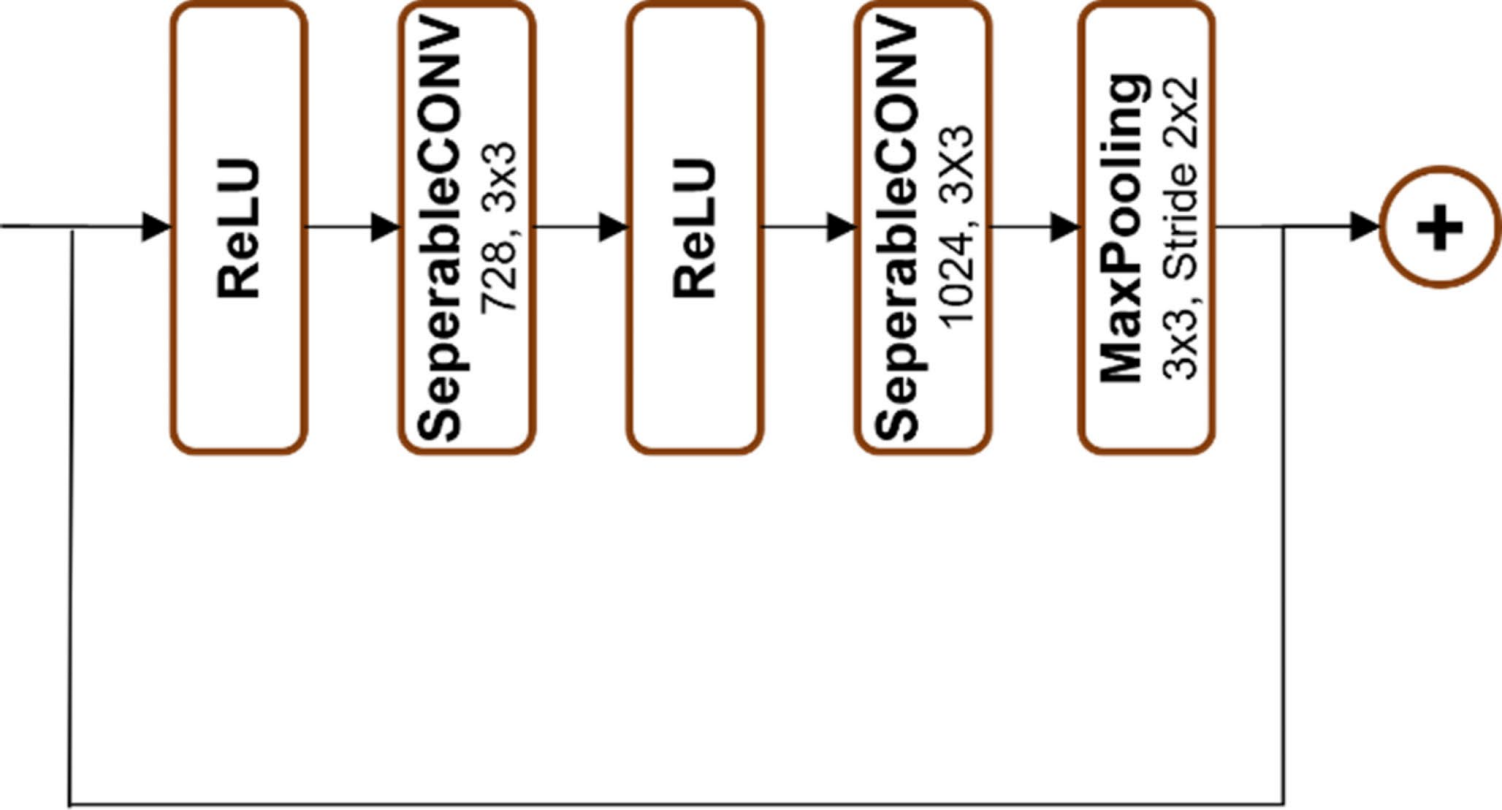
Block 13

**Fig. 6:**
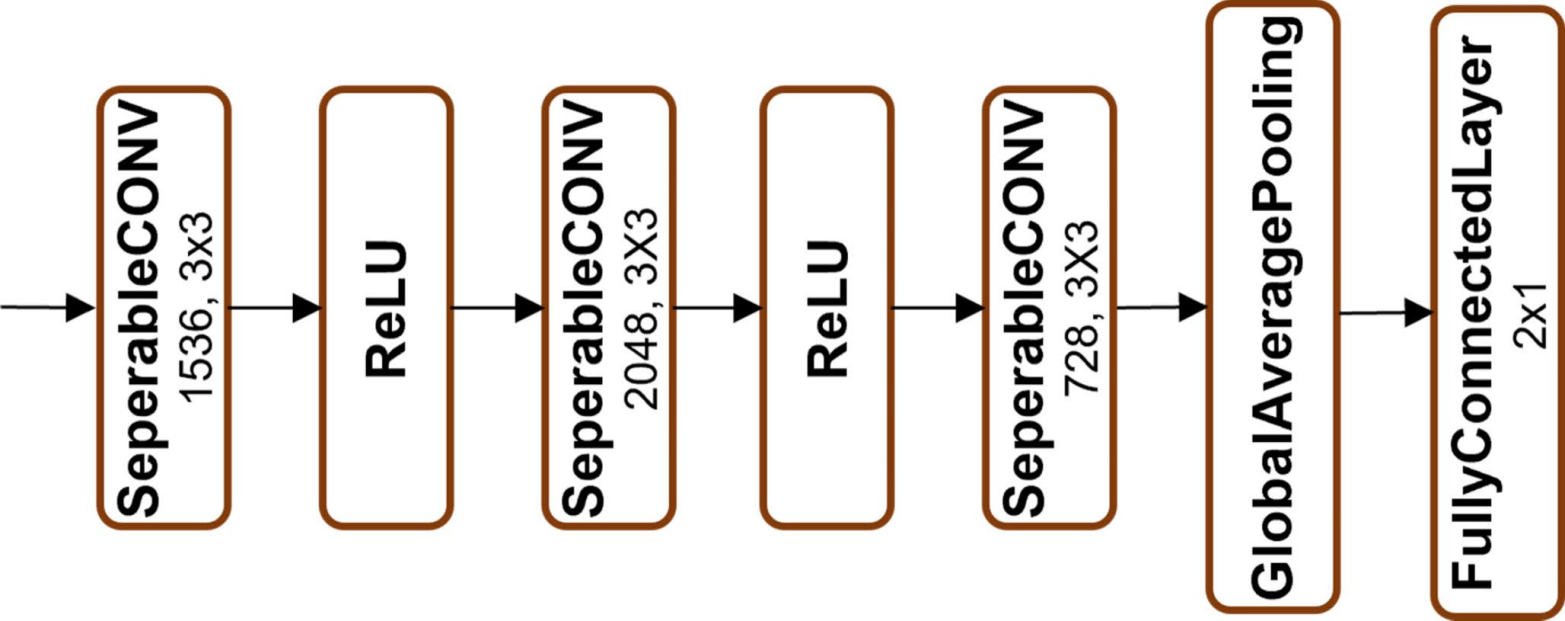
Block 14

As illustrated in Figs. 2, 3, 4, 5 and 6, Xception employs multiple separable convolution blocks with batch normalization and ReLU activation, forming a strong feature hierarchy. Like ResNet, skip connections improve gradient flow, enabling deeper networks. The model features entry and middle flows for extraction and refinement, followed by an exit flow where global average pooling and fully connected layers handle classification.

This efficient design suits resource-limited devices. Performance can be further enhanced through transfer learning, data augmentation, and hyperparameter tuning. Regularization, careful selection of loss functions, metrics, and batch normalization improve robustness. Additional techniques like pre-trained weights, ensembles, early stopping, cross-validation, and hardware optimization can yield even better results. These strategies make Xception a practical, high-performing option for advanced computer vision tasks.

**Fig. 7:**
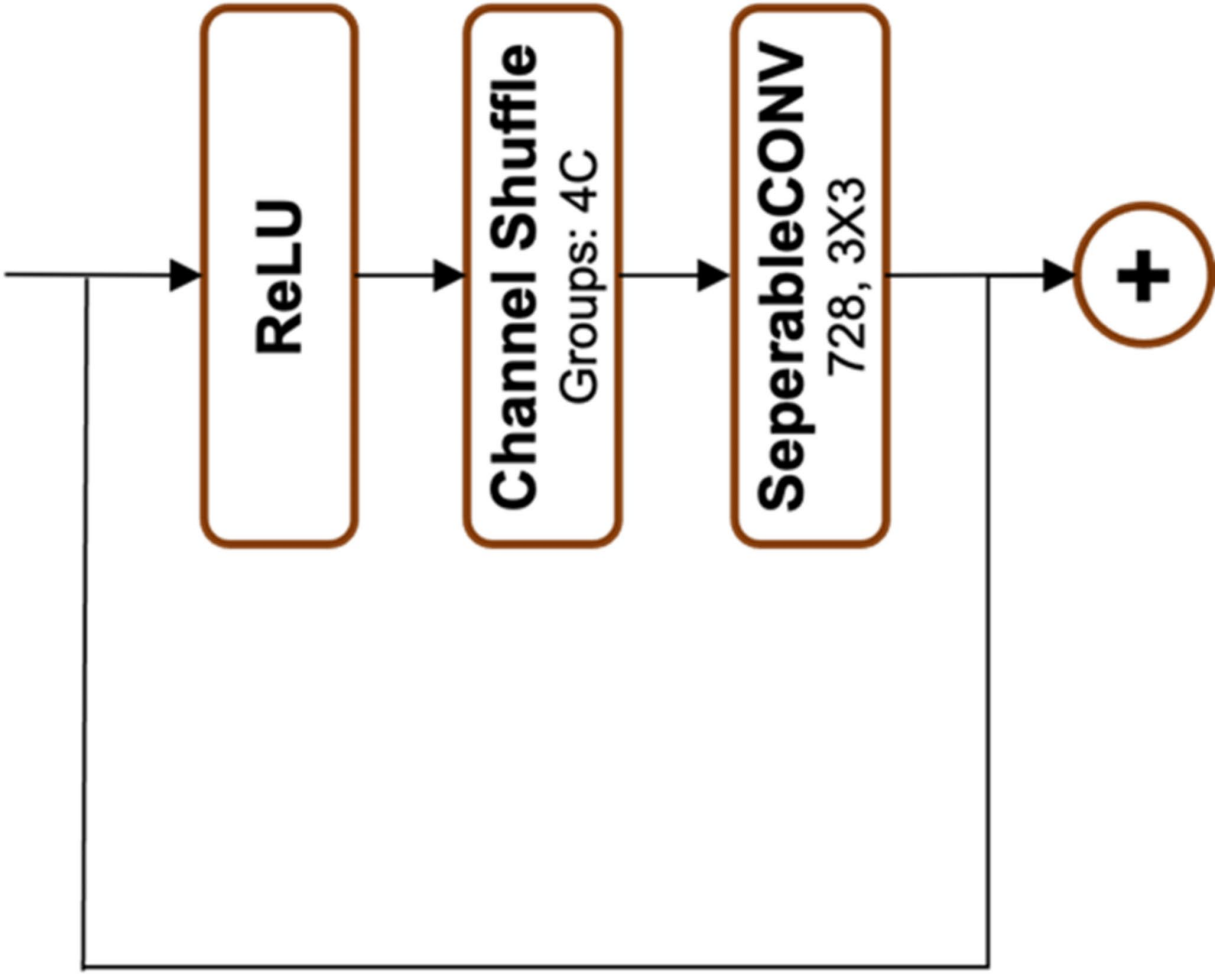
Shuffle Block

**Fig. 8:**
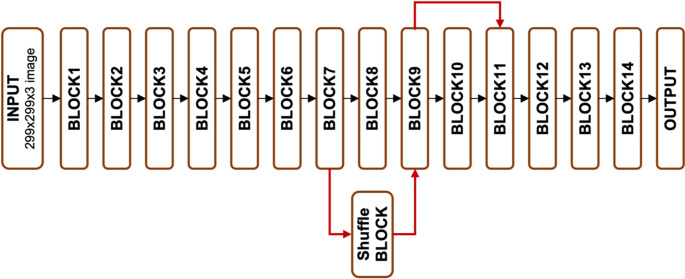
Forensic Xception model diagram

In this study, the Xception model’s performance is enhanced through a series of modifications described in the “Experiments & Results” section. The revised architecture, called “Forensic Xception,” incorporates two new residual connections. The first connection is introduced between the 7th and 9th blocks and includes a shuffle block, as illustrated in Fig. 7. The second connection is placed between the 9th and 11th blocks. A schematic diagram of the Forensic Xception model is presented in Fig. 8.

The Forensic Xception architecture integrates shuffle blocks within skip connections to facilitate information exchange while preserving spatial structure. Key features and implications include:

1. Feature Interaction: Integrating features from different layers encourages diverse information exchange and the capture of complex patterns.
2. Dimensionality Reduction: Shuffle blocks reorder channels to enhance channel-wise information flow, as seen in ShuffleNet [42] and Residual Channel Shuffled Attention Networks [45]. Combined with depthwise separable convolutions [39], which split standard convolutions into spatial and channel-specific operations, this approach improves efficiency while retaining spatial details.
3. Enhanced Representations: By promoting feature diversity and capturing a wide range of patterns, the model improves its generalization ability.
4. Regularization: Non-linearity introduced through depthwise separable convolutions and ReLU activations enables hierarchical feature learning [39, 46]. Stochasticity via dropout [47] prevents overfitting, leading to more robust feature representations.
5. Comparison with Xception: Unlike the original Xception, Forensic Xception employs shuffle blocks.
6. Implications: While Forensic Xception may enhance feature interactions and improve generalization, it likely introduces additional computational complexity compared to the original Xception.

The Forensic Xception architecture introduces shuffle blocks within skip connections to enhance feature interaction and representation learning. Before integrating this approach, we evaluated the performance of the model both with and without shuffle blocks. Including shuffle blocks resulted in improved computational efficiency and better performance on key metrics, due to their effective channel reorganization.

By incorporating shuffle blocks, the Forensic Xception model gains greater feature diversity and converges faster during training. This design enables it to capture complex patterns and relationships in the data, thereby enhancing generalization and robustness. However, these benefits come at the cost of increased computational complexity compared to original Xception.

### Polygon area metric

Evaluating classifier performance is challenging, especially with imbalanced datasets. Classification accuracy (CA), the most common metric, may not fairly represent performance across all classes. Other metrics like sensitivity (SE), specificity (SP), Kappa (K), area under the curve (AUC), and the F1 score (FM) address specific shortcomings but can complicate comparisons when used together.

To simplify this process, the Polygon Area Metric (PAM) was introduced [48]. PAM evaluates classifier performance by integrating multiple metrics—such as CA, SE, SP and FM into a single value. PAM computes the geometric area of a polygon formed by plotting these metrics on a normalized radar chart. A PAM value of 1 represents perfect and balanced classifier performance across all metrics, while a lower PAM value indicates inconsistent or suboptimal performance in one or more areas. This approach provides a more comprehensive assessment compared to single metrics, such as accuracy or the AUC scale, which primarily focus on specific trade-offs like SE and SP or precision and recall The proposed metric has been tested on seven different datasets using linear discriminant analysis, k-nearest neighbors and support vector machines classifiers, demonstrating its stability and validity.

## Experiments & results

This section reports the performance of various deep learning (DL) models on orthopantomography (OPG) images. The primary goal was to classify pediatric patients into two age categories: under 12, and 12 or older. We assessed models using PAM and established metrics like CA, SE, SP, K, AUC, and FM.

### Dataset description

**Fig. 9:**
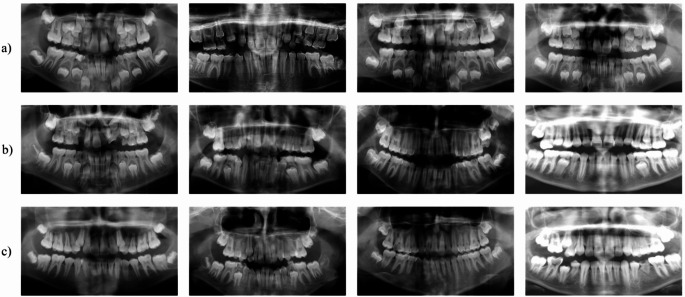
OPG images of children of different ages to be evaluated for forensic examinations. (**a**) Images of 7-year-old children, (**b**) Images of 11-year-old children, (**c**) Images of 12-year-old children

Our dataset included 1,941 pediatric patient OPG images, from patients aged 5 to 15 years, acquired using Orthopantomograph OP200D and Planmeca Proline XC devices at two radiology departments (Fig. 9). The dataset was divided into two different categories: 1,514 patients under the age of 12 and 427 patients over the age of 12.

### OPG image denoising

We used the Deep Convolutional Neural Network for Image Denoising (DnCNN) to improve OPG image quality. Operating on 50 × 50 grayscale patches, DnCNN removes noise while preserving crucial details. Unlike traditional methods, it learns directly from data, adapting to various noise patterns. This patch-based approach maintains local structures, enhancing the accuracy of subsequent classification tasks and ensuring clearer images for precise forensic examinations.

### Experimental setup

Initial experiments were conducted in MATLAB R2023b [49] on a setup with an Intel(R) i7 2.10 GHz processor, 32GB RAM, and an NVIDIA A2000 GPU. Final experiments ran on an Intel(R) Xeon(R) 2.60 GHz processor, 32GB RAM, and an NVIDIA T1200 GPU. These configurations supported efficient DL model training.

Given the class imbalance (1,514 images for under-12 vs. 427 for 12-and-older), we applied data augmentation by rotating images to create a balanced dataset. We then randomly divided data into training (70%) and testing (30%) sets. To ensure stability and reliability, each experiment was repeated ten times with randomized splits, allowing us to evaluate both mean performance and consistency across trials.

### Performance metrics

Following the classification experiments, we compute True Positive (TP), True Negative (TN), False Positive (FP), and False Negative (FN) values. These represent, respectively, the counts of correctly identified positives, correctly identified negatives, incorrectly identified positives, and incorrectly identified negatives. Based on these values, the following performance metrics are calculated:

CA: Measures the overall correctness of classification.

1 $$\:CA=\frac{TP+TN}{TP+TN+FP+FN}$$

SE: Known as the true positive rate, it evaluates the model’s ability to correctly identify positive instances.

2 $$\:SE=\frac{TP}{TP+FN}$$

SP: Indicates the model’s ability to correctly identify negative instances.

3 $$\:SP=\frac{TN}{TN+FP}$$

AUC: Assesses how well the model distinguishes between classes. It is obtained by integrating the Receiver Operating Characteristic (ROC) curve, which plots SE against (1–SP) for various thresholds.

4 $$\:AUC={\int\:}_{0}^{1}f\left(x\right)dx$$

K: Reflects agreement between predictions and true classes, accounting for chance.

5 $$\:K=\frac{TP}{TP+FP+FN}$$

FM: Combines precision and recall into a single measure, balancing false positives and false negatives.

6 $$\:FM=\frac{2TP}{2TP+FP+FN}$$

PAM: Provides a unified measure by integrating CA, SE, SP, AUC, K, and FM into a single value within a regular hexagon. To normalize PAM to the range [0, 1], the PA value is divided by 2.59807, which represents the area of the hexagon.

7 $$\:PAM=\frac{PA}{2.59807}$$

### Results

The average performance metrics for each DL model after ten experiments are summarized in Table 1. Additionally, Table 2 presents the standard deviations for these metrics, providing insights into the consistency and stability of the models’ performance. The following Table 3 shows a summary and comparison of the DL models evaluated.

**Table 1:** Average of performance metrics for each DL model after 10 experiments

Network	PAM	CA	SE	SP	AUC	K	FM
**Forensic Xception**	**0.8828**	**0.9400**	**0.9421**	**0.9328**	**0.9374**	**0.9245**	**0.9607**
Xception	0.8476	0.9174	0.9139	0.9297	0.9218	0.8960	0.9450
ResNet101	0.8394	0.9055	0.8899	0.9609	0.9254	0.8802	0.9363
ShuffleNet	0.8388	0.9196	0.9275	0.8914	0.9095	0.8999	0.9473
InceptionV3	0.8359	0.9093	0.9031	0.9313	0.9172	0.8858	0.9393
ResNet50	0.8280	0.9125	0.9194	0.8883	0.9038	0.8911	0.9417
DarkNet53	0.8255	0.9069	0.9062	0.9094	0.9078	0.8833	0.9376
InceptionResNetV2	0.8248	0.8974	0.8817	0.9531	0.9174	0.8699	0.9300
DarkNet19	0.8224	0.9069	0.9090	0.8992	0.9041	0.8836	0.9379
NasNetLarge	0.8196	0.8952	0.8808	0.9461	0.9135	0.8673	0.9283
NasNetMobile	0.8192	0.9045	0.9055	0.9008	0.9031	0.8806	0.9363
DenseNet201	0.8108	0.8933	0.8850	0.9227	0.9038	0.8664	0.9276
EfficientNetB0	0.8101	0.9034	0.9115	0.8750	0.8932	0.8800	0.9353
MobileNetV2	0.7954	0.8806	0.8645	0.9375	0.9010	0.8491	0.9176
ResNet18	0.7710	0.8581	0.8383	0.9281	0.8832	0.8218	0.8874
GoogleNet	0.7700	0.8761	0.8773	0.8719	0.8746	0.8455	0.9139
SqueezeNet	0.6383	0.7600	0.7211	0.8977	0.8094	0.6957	0.7729
AlexNet	0.4913	0.6971	0.7104	0.6500	0.6802	0.6349	0.7371

**Table 2:** Standard deviation of performance metrics for each DL model after 10 experiments

Network	PAM	CA	SE	SP	AUC	K	FM
**Forensic Xception**	**0.0131**	**0.0110**	**0.0185**	**0.0206**	**0.0057**	**0.0141**	**0.0076**
Xception	0.0246	0.0168	0.0268	0.0379	0.0145	0.0216	0.0122
ResNet101	0.0436	0.0329	0.0498	0.0441	0.0187	0.0424	0.0245
ShuffleNet	0.0210	0.0084	0.0201	0.0569	0.0206	0.0108	0.0060
InceptionV3	0.0236	0.0145	0.0296	0.0616	0.0207	0.0190	0.0107
ResNet50	0.0638	0.0402	0.0608	0.0980	0.0415	0.0515	0.0307
DarkNet53	0.0376	0.0289	0.0488	0.0659	0.0219	0.0375	0.0217
InceptionResNetV2	0.0343	0.0294	0.0475	0.0412	0.0110	0.0383	0.0221
DarkNet19	0.0401	0.0238	0.0484	0.1048	0.0361	0.0313	0.0180
NasNetLarge	0.0471	0.0364	0.0544	0.0403	0.0183	0.0470	0.0278
NasNetMobile	0.0258	0.0205	0.0418	0.0738	0.0219	0.0272	0.0155
DenseNet201	0.0696	0.0430	0.0490	0.0409	0.0383	0.0525	0.0315
EfficientNetB0	0.0593	0.0424	0.0677	0.0927	0.0354	0.0549	0.0330
MobileNetV2	0.0510	0.0408	0.0623	0.0483	0.0192	0.0530	0.0321
ResNet18	0.1880	0.1515	0.1930	0.0396	0.0989	0.1899	0.1572
GoogleNet	0.0907	0.6720	0.1076	0.1442	0.0542	0.0877	0.0559
SqueezeNet	0.2609	0.2364	0.3241	0.1374	0.1340	0.3053	0.2958
AlexNet	0.2015	0.2099	0.3257	0.3097	0.1178	0.2731	0.2655

**Table 3:** Summary of DL models evaluated

Network	Architecture	Key Features	Notes
Forensic Xception	Modified Xception	Shuffle block integration, skip connections	Outperformed all other models
Xception	Standard Xception	Depthwise separable convolutions	Baseline for comparison
ResNet101	Residual Network	Deep residual learning	Strong performance in specificity
ShuffleNet	Lightweight CNN	Pointwise group convolution, channel shuffle	Balanced performance across all metrics
InceptionV3	Inception	Multi-scale convolutional filters	Suitable for general-purpose classification
ResNet50	Residual Network	Deep residual learning, moderate depth	Strong performance in balanced tasks
DarkNet53	YOLO Backbone	Real-time detection	High speed, moderate accuracy
InceptionResNetV2	Hybrid Inception + ResNet	Combines multi-scale filters with residuals	High specificity, balanced performance
DarkNet19	YOLO Backbone	Simplified version of DarkNet53	Efficient for smaller datasets
NasNetLarge	Neural Architecture Search	Optimized for image recognition	High computational requirements
NasNetMobile	Mobile-Friendly NAS	Optimized for mobile applications	Compact design for mobile devices
DenseNet201	Dense Connectivity	Feature reuse to improve gradient flow	Memory-efficient architecture
EfficientNetB0	Optimized Network	Balances depth, width, and resolution	Well-suited for resource-constrained tasks
MobileNetV2	Mobile-Friendly CNN	Inverted residuals and depthwise convolutions	High efficiency with compact architecture
ResNet18	Residual Network	Shallow depth for lightweight tasks	Suitable for simpler classification tasks
GoogleNet	Inception	Multi-scale convolutional filters	Lower overall accuracy
SqueezeNet	Compact CNN	Fire modules for parameter efficiency	Best suited for edge devices
AlexNet	Shallow CNN	Early deep learning architecture	Low accuracy, outperformed by modern models

**Fig. 10:**
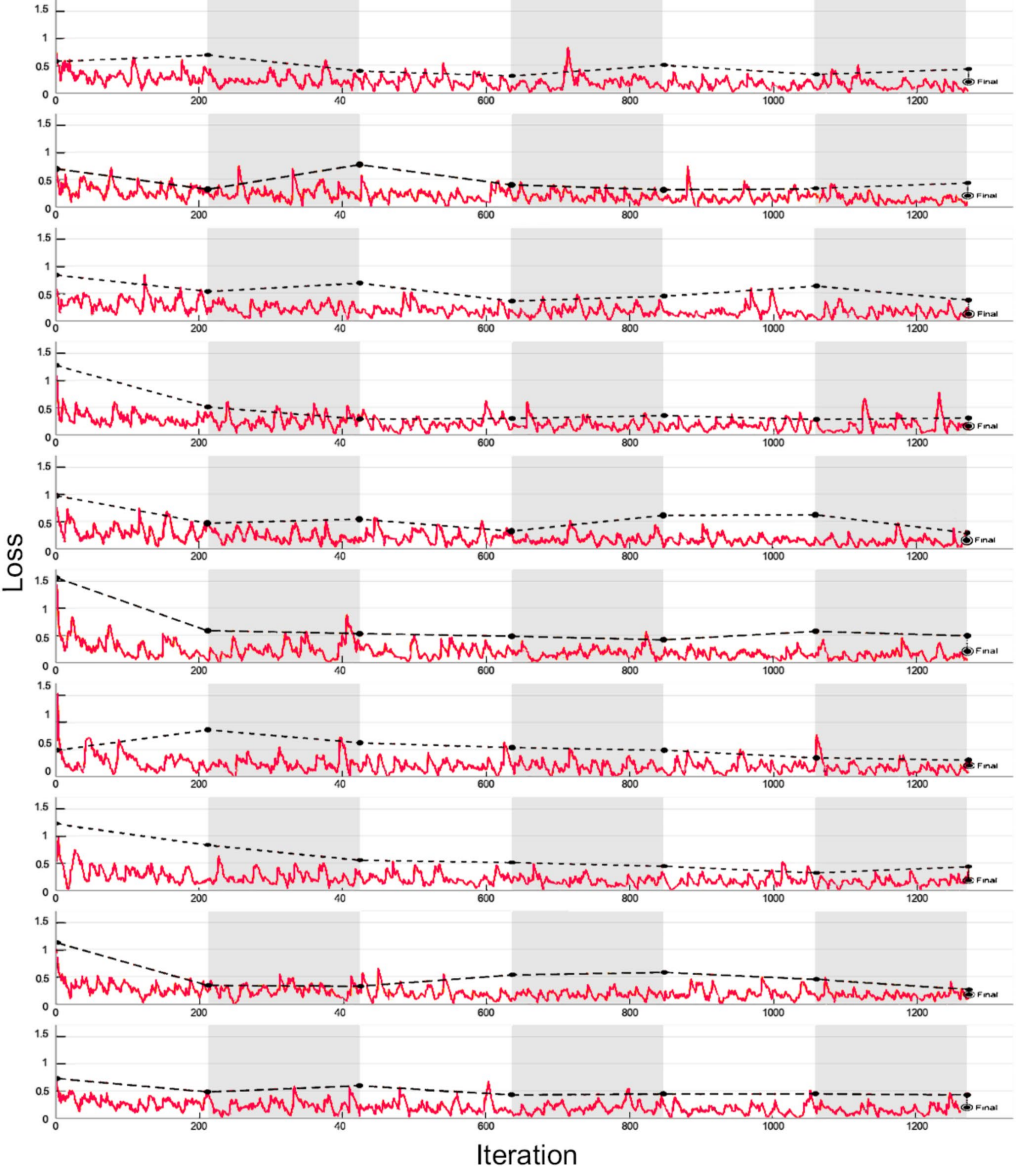
Loss graphs for 10 iterations of the Forensic Exception algorithm with 6 epochs. The graph tracks the decrease in loss values during training and validation phases, providing insight into the model’s learning behavior. Solid lines represents training loss and dashed lines represents validation loss of the model. A smooth decline in loss indicates effective optimization, while any significant divergence between training and validation loss may signal overfitting or issues with generalization

The Forensic Xception model’s loss function trajectories illustrate the complexity and variability of deep learning training (Fig. 10). The training loss reflects how well the model fits the training data, while the validation loss assesses the model’s ability to generalize to unseen data. A consistent decrease in both loss values, with minimal divergence, indicates successful optimization and generalization. These graphs also highlight any potential issues, such as overfitting, which would manifest as a growing gap between training and validation loss.

Despite varying initial loss values (0.5 to 1.5), all runs converge near 0.25 by training’s end, demonstrating the model’s capacity to learn despite differing starting conditions. This successful convergence confirms that the model effectively extracts meaningful features from OPG images and discriminates between individuals under 12 and those aged 12 or older, supporting its suitability for forensic dental age estimation.

Moreover, the training protocol’s consistency—each experiment finishing after 6 epochs and 1272 iterations—underscores the reproducibility of these findings, minimizing the effects of randomness and reinforcing confidence in the results.

**Fig. 11:**
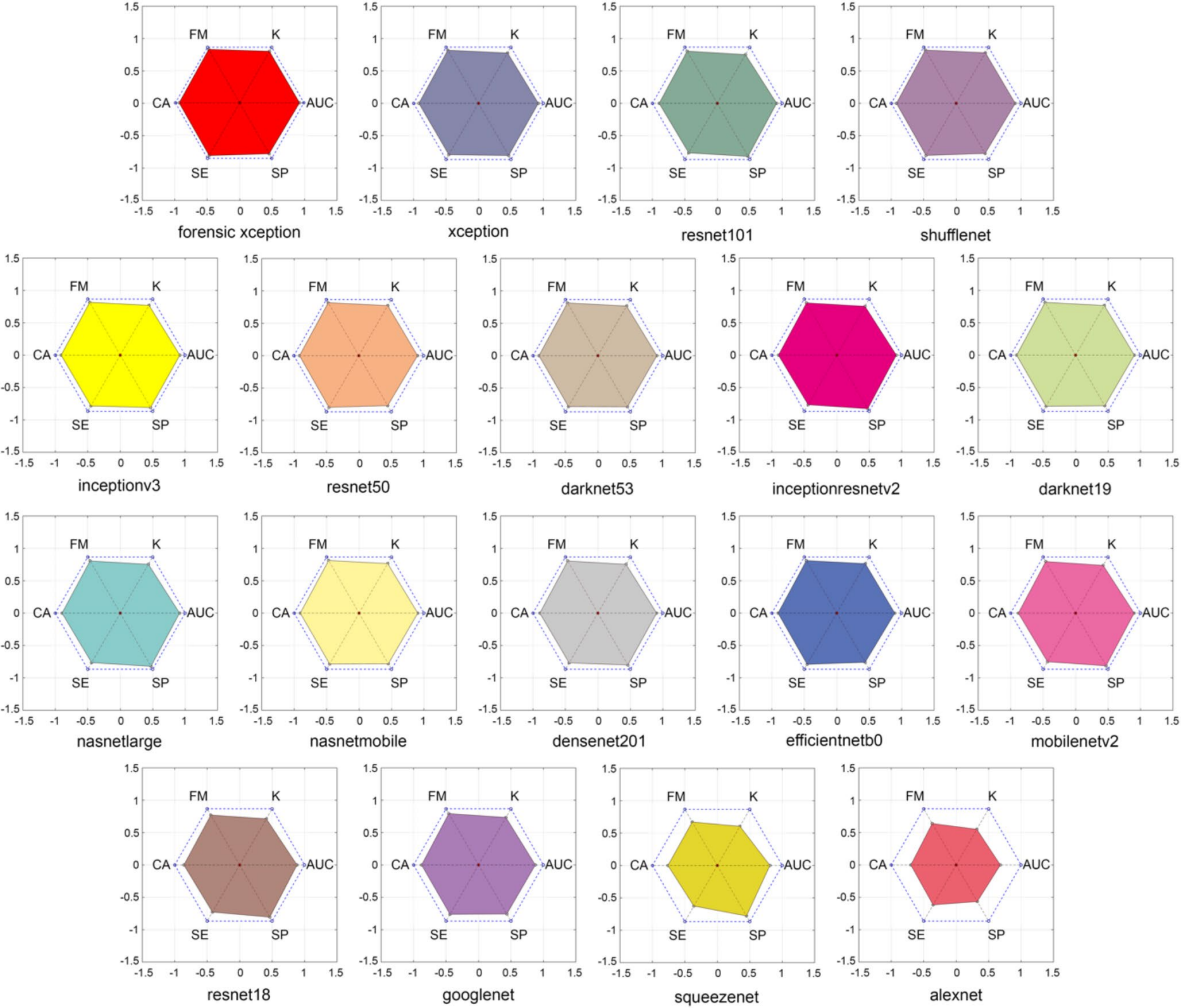
PAM of all DL models

Figure 11 presents radial graphs plotting CA, SE, SP, K, AUC, and FM for each model. The filled area within these radar charts corresponds to the PAM scores, enabling an immediate visual comparison of model performance across multiple metrics.

**Fig. 12:**
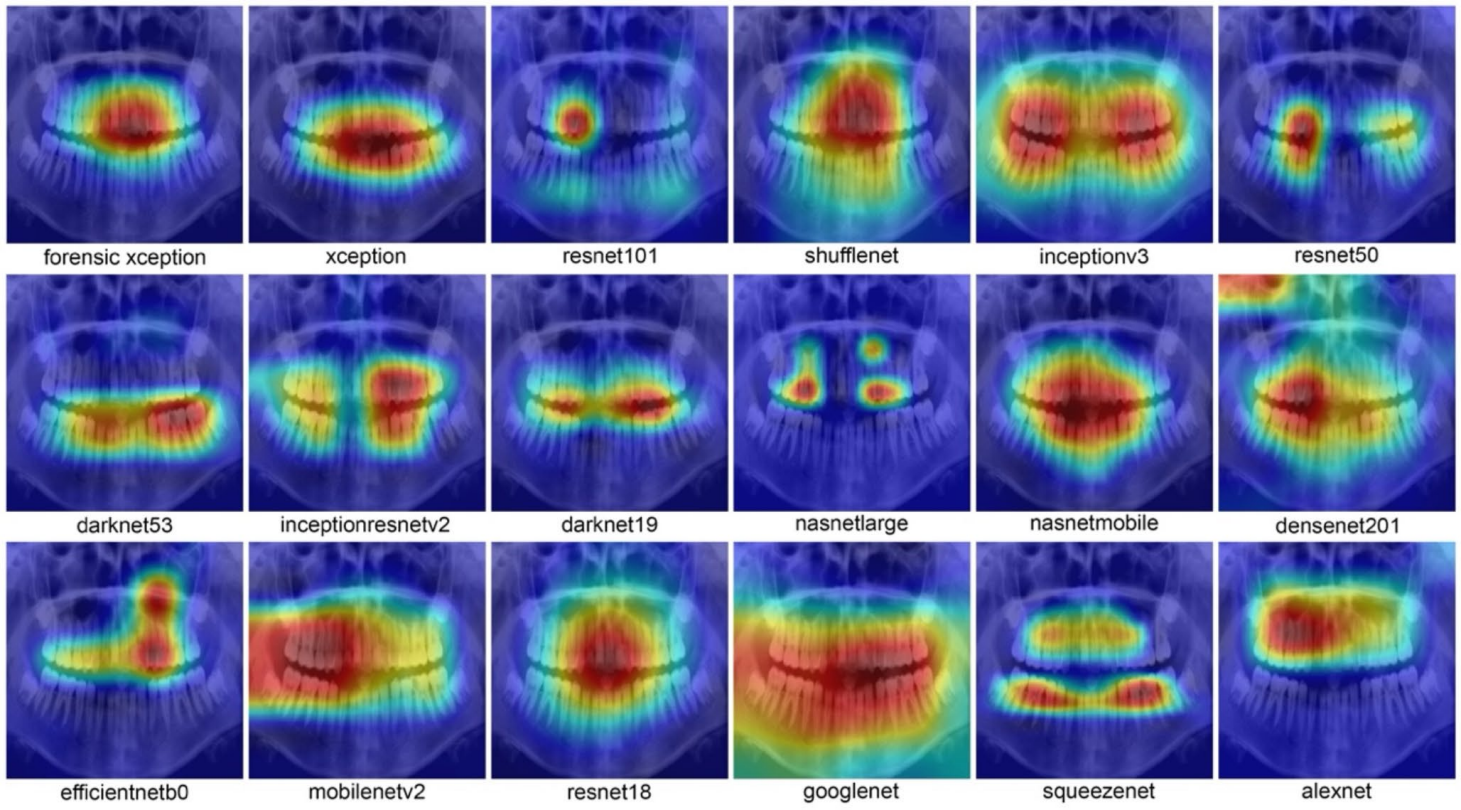
Heatmap of all DL models. The transitioning colored area, from red at the center to yellow at the edges, represents the considered features during classification

Figure 12 offers a heatmap visualization of each model’s focus within a sample OPG image. The red-to-yellow gradient highlights the regions the model prioritizes during classification. Such visualization is vital for interpretability, guiding improvements and refinements in future model iterations.

## Discussion and conclusion

In legal contexts, accurate forensic age estimation is critical for determining criminal responsibility, especially for individuals under 12. This study evaluates deep learning (DL) models on OPG images and identifies the “Forensic Xception” model as a standout performer. It achieves a PAM score of 0.8828 and consistently excels across CA, SE, SP, AUC, K, and FM metrics, underscoring its forensic value.

Evaluating the replicability and generalizability of the Forensic Xception model involved testing it on previously unseen samples. Consistent results across these samples confirmed the model’s reliability in maintaining stable predictions under similar conditions.

Heatmaps generated by the Forensic Xception model offer valuable insights into its interpretability and areas for improvement. These visualizations highlight image regions that most influenced the model’s decisions. For age estimation, they consistently emphasized dental structures like molars and incisors, confirming their relevance in determining age. Interpreting these highlighted regions helps validate predictions, ensure alignment with expert knowledge, and identify potential improvements. If heatmaps occasionally emphasize irrelevant areas, it may indicate noise or non-informative features affecting the model’s generalizability.

By examining these patterns, forensic experts can understand the model’s decision boundaries, spot biases, and guide refinements. This approach aligns with the broader goal of making deep learning more transparent and explainable.

While the Forensic Xception model shows promise in automated forensic age estimation—especially for individuals near the critical age cutoff of 12—selecting the ideal model depends on specific needs and constraints. Different forensic applications may weigh performance metrics differently. The proposed tool streamlines analysis, saving time and effort while improving consistency in age classification.

As new data emerges, retraining can enhance accuracy and adaptability. Planned deployments include user-friendly interfaces with interpretability features like heatmaps, ensuring experts can understand the model’s reasoning. Collaboration with forensic institutions and adherence to ethical and legal standards will support its reliability in real-world applications.

Still, this study’s scope is limited to a specific dataset. Broader validation on diverse populations is needed to confirm its general applicability. Future efforts include external validation on various datasets, applying transfer learning for different forensic scenarios, and ensuring replicability across contexts.

Continuing research with diverse datasets, fine-tuning models, and addressing ethical implications will help align these advancements with the justice system’s standards and requirements.

## Key Points

1.  “Forensic Xception” proposed to improve forensic age estimation of juveniles from orthopantomography (OPG) images.
2.  Various deep learning architectures were assessed to identify the most effective model for the task.
3.  “Forensic Xception” outperformed other models with a PAM score of 0.8828.
4.  This model sets a precedent for further advancements in forensic dental age estimation.
